# Antisense oligonucleotide inhibition of Heat Shock Protein (HSP) 47 improves bleomycin-induced pulmonary fibrosis in rats

**DOI:** 10.1186/1465-9921-8-37

**Published:** 2007-05-15

**Authors:** Satoshi Hagiwara, Hideo Iwasaka, Shigekiyo Matsumoto, Takayuki Noguchi

**Affiliations:** 1 Department of Brain and Nerve Science, Anesthesiology, Oita University Faculty of Medicine, 1-1 Idaigaoka-Hasamamachi-Yufu City-Oita 879-5593, Japan

## Abstract

**Background:**

The most common pathologic form of pulmonary fibrosis arises from excessive deposition of extracellular matrix proteins such as collagen. The 47 kDa heat shock protein 47 (HSP47) is a collagen-specific molecular chaperone that has been shown to play a major role during the processing and/or secretion of procollagen.

**Objectives:**

To determine whether inhibition of HSP47 could have beneficial effects in mitigating bleomycin-induced pulmonary fibrosis in rats.

**Methods:**

All experiments were performed with 250–300 g male Wistar rats. Animals were randomly divided into five experimental groups that were administered: 1) saline alone, 2) bleomycin alone, 3) antisense HSP47 oligonucleotides alone, 4) bleomycin + antisense HSP47 oligonucleotides, and 5) bleomycin + sense control oligonucleotides. We investigated lung histopathology and performed immunoblot and immunohistochemistry analyses.

**Results:**

In rats treated with HSP47 antisense oligonucleotides, pulmonary fibrosis was significantly reduced. In addition, treatment with HSP47 antisense oligonucleotides significantly improved bleomycin-induced morphological changes. Treatment with HSP47 antisense oligonucleotides alone did not produce any significant changes to lung morphology. Immunoblot analyses of lung homogenates confirmed the inhibition of HSP47 protein by antisense oligonucleotides. The bleo + sense group, however, did not exhibit any improvement in lung pathology compared to bleomycin alone groups, and also had no effect on HSP47 expression.

**Conclusion:**

These findings suggest that HSP47 antisense oligonucleotide inhibition of HSP47 improves bleomycin-induced pulmonary fibrosis pathology in rats.

## Introduction

Pulmonary fibrosis results from various lung injuries and is a potentially lethal disorder with no effective therapies currently. Alveolar wall fibrosis with an accumulation of extracellular matrix molecules, notably collagen, is characteristic [1]. The tendency for progression to end-stage pulmonary disease has been correlated to the degree of extracellular matrix accumulation in the alveolar wall. Some studies have shown that fibrotic lesions contain the normal constituents of extracellular matrix, including collagen type IV and V, laminin, and interstitial (type I and III) collagens [2,3]. However, the underlying molecular mechanisms responsible for excessive deposition of collagen in fibrotic lesions are not fully understood.

Collagens constitute a family of extracellular proteins that begin their assembly within the endoplasmic reticulum lumen and are subsequently secreted from the cell. Type I to V collagens have been shown to bind to a 47 kDa heat shock protein (HSP47) [4,5]. Accumulation of HSP47 plays a specific role as a molecular chaperone in the processing of procollagen molecules [6,7]. HSP47 binds specifically to collagen and not to other proteins such as fibronectin or laminin [8].

Under pathologic conditions, HSP47 expression is associated with collagen biosynthesis in carbon tetrachloride (CCl_4_)-induced rat liver fibrosis [9], anti-thymocyte serum induced glomerulonephritis [10], and a rat remnant kidney model [11]. These findings suggest that HSP47 may play a crucial role in collagen accumulation. Antisense phosphorothioate oligonucleotides may be a potential experimental and therapeutic tool for downregulating HSP47. Hence, this study aimed to examine whether HSP47 downregulation can suppress collagen accumulation in an animal model of bleomycin-induced pulmonary fibrosis. We also explored whether HSP47-antisense could be a potential therapeutic application for pulmonary fibrosis.

## Materials and methods

### Animals

All protocols conformed to the National Institute of Health (NIH) guidelines, and animals received care in compliance with the Principals of Laboratory Animal Care. Male Wistar rats weighing 250–300 g (Kyudou, Saga, Japan) were used in all experiments. All animals were housed with free access to food and water ad libitum.

### Experimental protocols

Animals were randomly assigned to one of the five groups as follows: 1) control group (n = 20) with an intratracheal administration of 0.9% NaCl solution alone (saline); 2) bleomycin group (n = 20) with an intratracheal administration of bleomycin (Sigma, St. Louis, MO) dissolved in 0.9% NaCl solution (10 unit/kg) (Bleo); 3) antisense group (n = 20) with an intratracheal administration of antisense oligonucleotides dissolved in 0.9% NaCl solution (100 nmol/kg) alone (AS); 4) bleomycin + antisense group (n = 20) with an intratracheal administration of antisense oligonucleotides dissolved in 0.9% NaCl solution (100 nmol/kg) with bleomycin (10 unit/kg) (Bleo+AS); and 5) bleomycin + sense control group (n = 4) with an intratracheal administration of sense control oligonucleotides dissolved in 0.9% NaCl solution (100 nmol/kg) with bleomycin (10 unit/kg) (Bleo+S).

All rats were anesthetized with 3% sevofluorane. The trachea was exposed through a midline anterior neck incision and each agent was injected into the trachea using a 24-gauge needle. At 14 or 28 days after surgery, animals were sacrificed by clipping the vena cava and transecting the aorta, followed by an intra-atrial injection of normal saline. Lung tissue was quickly removed and processed as described below.

### HSP47 antisense and sense control oligonucleotides

Antisense phosphorothioate oligonucleotides (Biognostik GmbH, Germany) were designed as 15 mer analogues targeted to the first five codons of HSP47. In addition, we created sense control oligonucleotides that did not act on HSP47. The sequences of the phosphorothioate oligonucleotides were as follows:

HSP47 antisense: 5'-TACGCGAGAGAGGAA-3'

Sense control: 5'-GTCCCTATACGAACG-3'

These phosphorothioates oligonucleotides were more stable *in vivo *and more resistant to intracellular enzymes when compared with natural phosphodiesters [12].

### Histological analysis

Right lower lobe lungs were obtained from animals and instilled with 10% formalin. Samples were embedded in paraffin and then cut into 4 μm thickness sections. Sections were stained with hematoxylin and eosin (H&E), and the severity of interstitial fibrosis among the groups was compared using the Ashcroft score [13]. Azan staining was performed to visualize the collagen fibrils in the tissue.

### Hydroxyproline measurement

The total collagen content of the right lung was determined by hydroxyproline measurement. The hydroxyproline content was determined after acid hydrolysis of the right lung with 12 N HCl in a sealed glass tube (Iwaki, Tokyo, Japan) at 100°C for 20 hrs [14]. Data are expressed as milligrams of hydroxyproline per gram of protein in lungs (mg/g).

### Immunohistochemistry analysis of HSP47 protein

The lungs were obtained from each group animals at 14 and 28 days under sevofluran anesthesia. Tissue samples were fixed immediately in 4% paraformaldehyde at 4°C overnight, embedded in Optimal Cutting Temperature compound (Sakura Finetechnical Co., Tokyo, Japan), and cut into 5 μm sections. Endogenous peroxidase activity was blocked with 0.3% H_2_O_2 _and sodium azide (1 mg/ml) for 10 min. Non-specific protein binding was blocked by incubation in 10% sheep serum for 10 min. Sections were then incubated with anti-HSP47 monoclonal antibody (1:1000 dilution) overnight at 4°C (Stressgen Biotechnology, Temecula, CA). Sections were rinsed 3 times for 5 min each in PBS before incubation with peroxidase-conjugated anti-mouse IgG (1:1000 dilution) for 1 hrs and rinsed again with PBS 3 times for 5 min each. Slides were stained using the biotin avidin peroxidase complex system from an LSAB2 kit (Dako, Via Real Carpinteria, CA). After development, slides were counterstained with Mayer's hematoxylin.

### Immunoblot analysis of HSP47 protein

The lungs were obtained from animals after 14 and 28 days after a 10 unit/kg bleomycin or bleomycin + antisense oligonucleotide administration under sevofluran anesthesia. Blood was cleared by saline perfusion via the right ventricle and lungs were homogenized with a polytron homogenizer (IKA Labortechnik, Staufen, Germany) in a T-PER Tissue Protein Extraction Reagent (Pierce, Rockford, IL). After homogenization, the sample was centrifuged at 10,000 rpm for 5 min. Supernatant was measured for protein content using the BCA Protein Assay Regent (Pierce, Rockford, IL) at an absorbance of 562 nm.

Electrophoresis was performed using a discontinuous Tris-glycine buffer system. Proteins were suspended in sodium dodecyl sulfate polyacrylamide gel electrophoresis (SDS-PAGE) buffer (20 mM Tris-HCl, 0.2 M Glycine) and boiled for 1 min before loading onto 10% gels. Gels were immediately electrotransferred to polyvinilidene difluoride (PVDF) membranes (Millipore, Bedford, Massachusetts) at 60 V for 3 hrs by means of a wet transfer system (transfer buffer: 20 mM Tris-HCl, 0.2 M Glycine, 20% MeOH). Membranes were blocked with 5% nonfat dry milk in TBS/Tween buffer (25 mM Tris-HCl, 0.14 M NaCl, 2% Tween20) (Bio-Rad Lab., Hercules, CA) overnight, at 4°C. They were subsequently incubated with pre-immune serum, diluted 1:1000 in 1% nonfat dry milk, for 1 hr with gentle shaking at room temperature. Secondary antibody was also diluted in 1% nonfat dry milk (1:1000 dilution) and applied to the membrane for 1 hr at room temperature. Washing between and after antibody incubation steps was performed three times for 10 min each with TBS/Tween buffer. Proteins were revealed by enhanced chemiluminescence (ECL) (Amersham, Buckinghamshire, England) and exposed to film (Hyperfilm ECL; Amersham, Buckinghamshire, England). The film was scanned and protein band concentrations quantified by integrated optical density using NIH ImageJ software (National Institute of Health, U.S.A.).

### Statistical analysis

All data were presented as the mean ± standard error of the mean (S.E.M.). The data was analyzed by a statistical analysis of variance, a Scheffe's post-hoc test of group pairs for multiple comparisons, and an unpaired t-test for single comparisons. A p value < 0.05 was considered to be statistically significant.

## Results

### Antisense oligonucleotides reduce HSP47 protein

**Figure 1:**
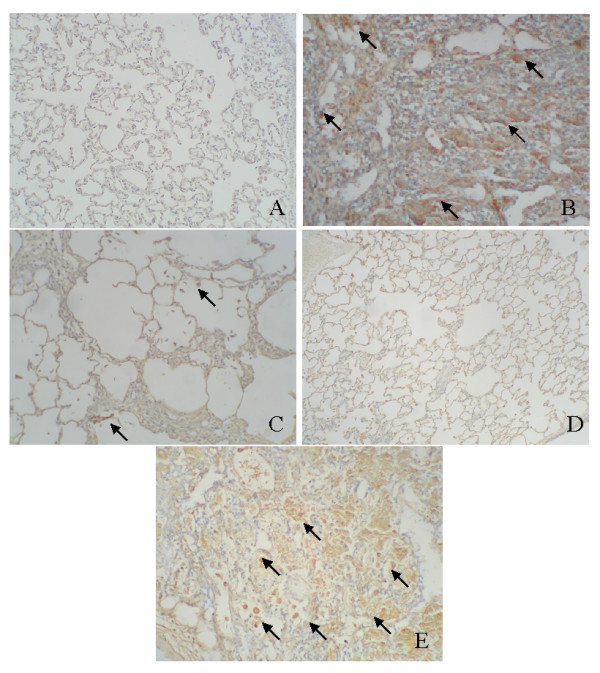
Immunohistochemistry for the detection of HSP47 in rat lungs treated with bleomycin with or without antisense HSP47 oligonucleotides (ODN). (A) A representative lung from a control rat sacrificed at 4 weeks after saline administration; 400×. (B) A representative lung from an animal in the Bleo group and sacrificed at 4 weeks; 400×. (C) A representative lung from an animal in the Bleo+AS group sacrificed at 4 weeks; 400×. (D) A representative lung from an animal in the antisense group sacrificed at 4 weeks; 400×. (E) A representative lungs from an animal in the Bleo+S group and sacrificed at 4 weeks; 400×. Arrows indicate immunopositivity cells to HSP47.

**Figure 2:**
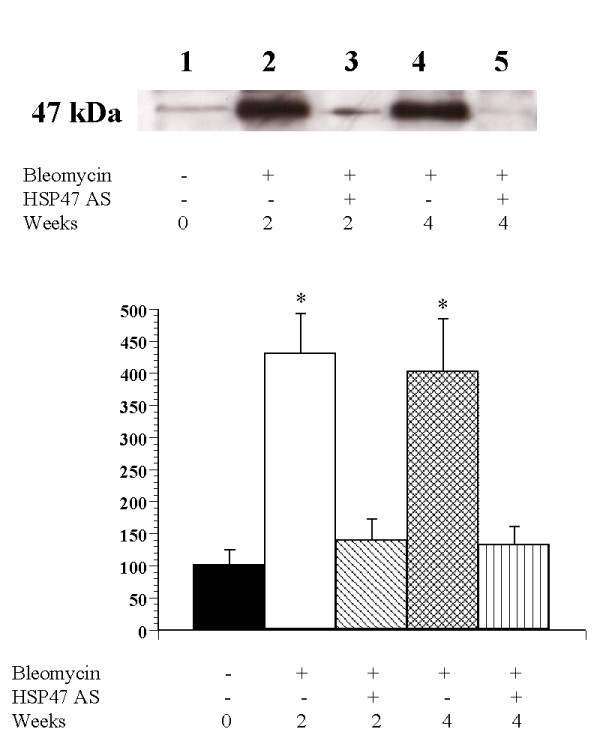
(A) Rat lungs treated with bleomycin was lysed and immunoblotted for HSP47 protein. Levels of HSP47 protein at 2 and 4 weeks were analyzed using Western blotting as described. HSP47 was up-regulated in the Bleo group compared to the Bleo+AS group. (B) HSP47 signal intensities were quantified using an image analyzer based on immunoblot density. HSP47 expression is shown as a percentage of the control. Data is expressed as mean ± SEM. * p < 0.05 vs. control group.

We examined levels of HSP47 protein in whole tissue and in thin sections of lungs from animals co-treated with bleomycin and sense or antisense oligonucleotides. Immunohistochemical analysis of thin sections was performed to examine the effect of antisense oligonucleotide treatment on bleomycin-induced HSP47 production *in vivo*. The Bleo (Fig. 1B) and Bleo+S (Fig. 1E) groups exhibited strong immunopositivity for HSP47. In contrast, HSP47 staining was significantly reduced in the saline, AS, and Bleo+AS groups (Fig. 1A, 1C, and 1D). Taken together, these data indicate that antisense oligonucleotides to HSP47 inhibit bleomycin-induced HSP47 production. Next, we utilized immunoblot analysis to examine the level of HSP47 protein inhibition in the Bleo+AS group as compared to the Bleo group. Increased levels of HSP47 protein were observed in lung tissue homogenates 2 and 4 weeks after bleomycin injection. Treatment with antisense oligonucleotides, however, inhibited the bleomycin-induced increase of HSP47 at both time points (Fig. 2A and 2B).

### Pathology

**Figure 3:**
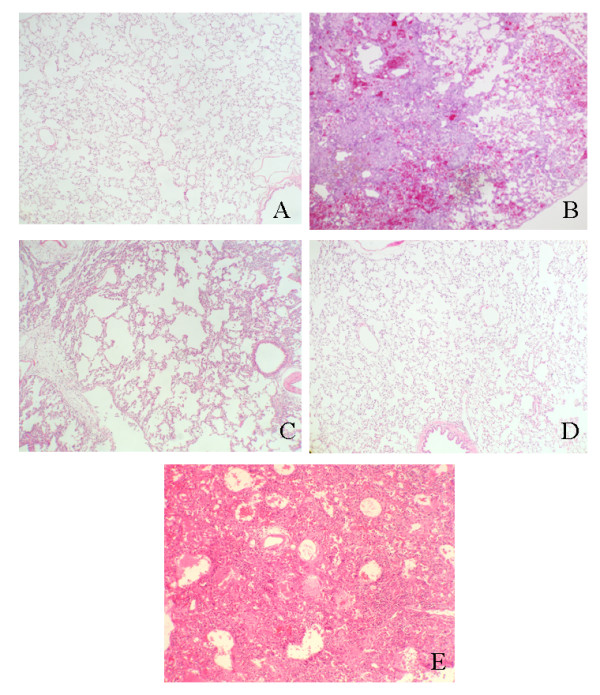
H&E staining of bleomycin-treated rat lungs with and without antisense HSP47 ODN. (A) Lungs from the saline group; 40× (B) Lungs from the Bleo group at 4 weeks following bleomycin treatment exhibit diffuse consolidation of parenchyma with loss of alveolar architecture and increased cell number; 40×. (C) Lungs from the Bleo+AS group at 4 weeks post-injection demonstrates improvement of lesions compared to the Bleo alone group; 40×. (D) Lungs from the AS alone group at 4 weeks post-injection show no significant pulmonary consolidation or fibrosis; 40×. (E) Lungs from the Bleo+S group at 4 weeks post-injection exhibit diffuse consolidation of parenchyma with loss of alveolar architecture and increased cell number; 40×.

**Figure 4:**
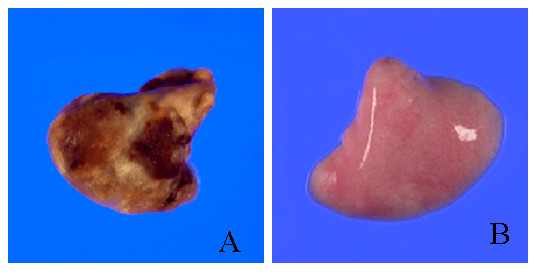
Macrograph of lungs treated with bleomycin alone (A) or bleomycin + antisense HSP47 ODN (B) and sacrificed 28 days later. (A) Lungs from a Bleo only treated animal exhibit fibrosis and loss of alveolar architecture. (B) Lungs from a Bleo+AS treated animal exhibit normal morphology and no signs of fibrosis.

**Figure 5:**
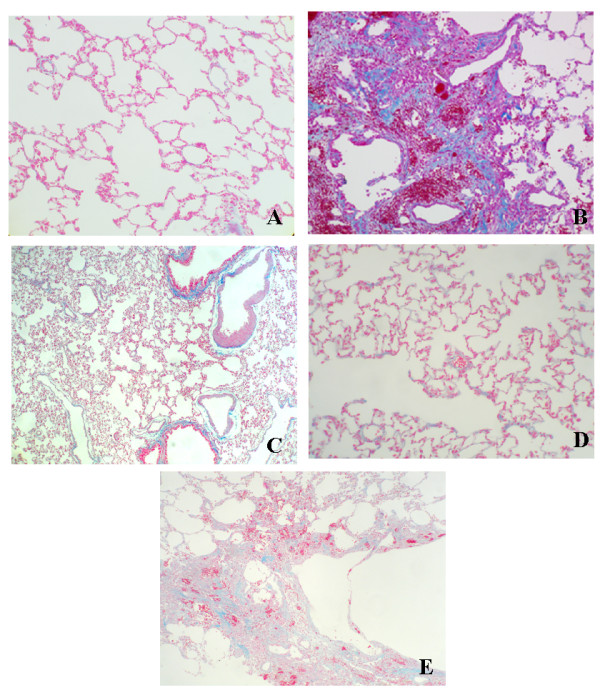
Azan staining of lung tissue from each group. (A) Azan staining of the saline group shows minimal collagen content in the lung parenchyma; 100×. (B) Azan staining of the Bleo group shows an increase in the collagen content of lung parenchyma; 100×. (C) Azan staining the Bleo+AS group shows a reduction in collagen fibrils compared to the saline and Bleo groups; 100×. (D) Azan staining the AS group shows minimal collagen in the lung parenchyma; 100×. (E) Azan staining of the Bleo+S group shows an increase in the collagen content of lung parenchyma, comparable to the Bleo group; 100×.

**Figure 6:**
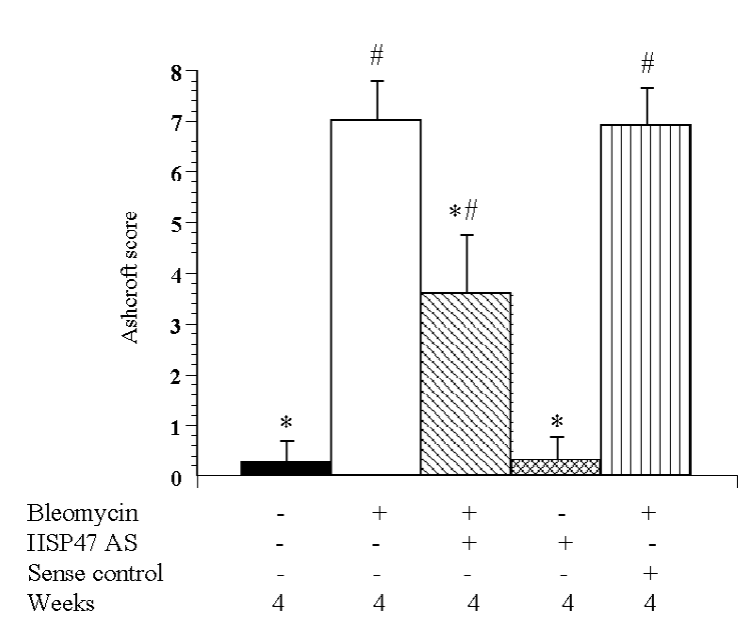
Histopathologic assessment of pulmonary fibrosis by the Ashcroft score. Comparison of lung tissue extracted at day 28 between the control, Bleo, Bleo+AS, AS, and Bleo+S groups (n = 8). Data are expressed as mean ± SEM. * p < 0.05 vs. Bleo+AS group; # p < 0.05 vs. control group.

Lungs were analyzed 4 weeks after treatment with bleomycin alone or bleomycin and antisense oligonucleotides. The lungs of the Bleo group rats exhibited acute alveolar injury and pneumonitis which progressed to severe interstitial and intra-alveolar pneumonia and/or fibrosis. In addition, we observed consolidation of the parenchyma with loss of the alveolar architecture and increased cell number. Such lesions varied from focal to diffuse, and collagen was clearly elevated (Fig. 3B). On the other hand, the Bleo+AS group demonstrated inhibition of pulmonary fibrosis (Fig. 3C). Lung sections from both the saline (Fig. 3A) and AS groups (Fig. 3D) did not show significant pulmonary consolidation or fibrosis when sacrificed at 4 weeks. The Bleo+S group demonstrated pulmonary fibrosis to the same extent as that of the Bleo group (Fig. 3E). In a macrograph, the lungs of the Bleo group were firm and lost significant elasticity (Fig. 4A). However, the lungs of the Bleo+AS group were almost completely normal (Fig. 4B). Azan stained sections showed increased connective tissue in the lungs of the Bleo group (Fig. 5B) compared with that of the Bleo+AS group (Fig. 5C). The saline and AS groups showed minimal amounts of collagen content in the lung parenchyma (Fig. 5A and 5D). On the other hand, the Bleo+S group showed an increase in the collagen content of lung parenchyma similar to the Bleo group (Fig. 5E). In addition, Ashcroft scores in the Bleo+AS group were significantly reduced compared to the Bleo group (Fig. 6).

### Mortality

Approximate thirty percent (30%) of bleo group rats died within 14 days after the administration of bleomycin, while all rats receiving with an intratracheal administration of antisense oligonucleotides with bleomycin survived. Kaplan-Meier analysis revealed a significantly shorter time-to-death among the bleo+AS group than the bleo group. All of the saline and AS group animals were survived through 28 days.

### Hydroxyproline content in lung tissue

**Figure 7:**
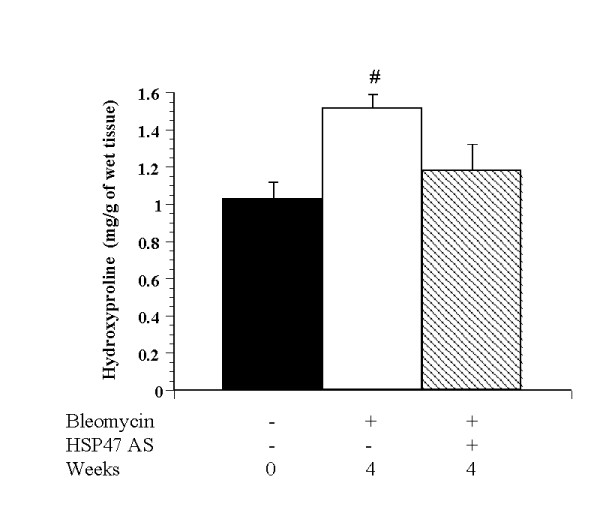
Hydroxyproline content of lung tissues (mg/g of wet tissue) at 28 days post Bleo injection. Comparison between control, Bleo, and Bleo+AS groups (n = 8). Data are expressed as the means ± SEM. # p < 0.05 vs. control group.

Collagen content of bleomycin-injected lungs was assessed by the hydroxyproline assay. The concentration of hydroxyproline in the lung tissue 4 weeks after bleomycin injection was significantly higher in the Bleo group than in the saline control group (Fig. 7). In the Bleo+AS group, hydroxyproline content was significantly reduced when compared with that in the Bleo group. There was no significant difference in hydroxyproline content in the AS group compared to controls (data not shown).

## Discussion

Pulmonary fibrosis is a common consequence, and often a central feature, of many lung diseases. It is also a highly lethal disorder. However, the pathogenesis of pulmonary fibrosis is poorly understood. Recently, various studies have examined the mechanisms of pulmonary fibrosis [15,16]. These mechanisms suggest that normal functional lung tissue is replaced with an abnormal accumulation of fibroblasts and collagen. As a result, pulmonary functions exhibit restricted volumes and capacities, restricted volumes, and decreased gas exchange [17]. Hence, collagen deposition must be controlled in order to reverse pulmonary fibrosis and improve the mortality rate.

HSP47, a 47 kDa collagen-binding glycoprotein, has the ability to function as a molecular chaperone in the endoplasmic reticulum [18]. It is involved in the processing and transporting of procollagen in the endoplasmic reticulum, and has a molecular chaperone-like function under stress conditions. The expression of HSP47 is always correlated with collagen expression *in vitro *[19].

It has been found that under pathologic conditions, the expression of HSP47 is significantly upregulated in conjunction with collagen expression during the progression of fibrosis in a rat liver fibrosis model [9] and a rat glomerulonephritis model [10]. It has also been shown that HSP47 typically colocalized with type I collagen in these models. These observations suggest that HSP47 might play an important role in the synthesis, processing, and secretion of procollagen [19]. In this study, expression of the HSP47 protein in the lung was increased during bleomycin-induced pulmonary fibrosis. These results suggest that HSP47 is expressed during pulmonary fibrosis.

HSP47 is the only chaperone protein characterized by specificity for collagen, and therefore, presents an attractive target for inhibiting disease progression. Antisense oligonucleotide of HSP47 has been shown to reduce the level of type I procollagen chains *in vitro *[20]. In the present study, we showed that collagen accumulation and disease progression in an experimental pulmonary fibrosis model were associated with the level of HSP47 protein expression. In this model, when rats were treated intratracheally by direct introduction of antisense oligonucleotides targeting HSP47, fibrotic lesions and collagen expression were significantly attenuated in line with a decrease in HSP47 expression. These findings support the theory that HSP47 plays an important role in the pathogenesis of pulmonary fibrosis.

Current therapies for pulmonary fibrosis utilizing corticosteroids and/or immunosupressants have shown little benefit [21]. Clinical trials with novel drugs based on these results are currently being investigated in various trial phases. However, to date, there are no effective therapies for treating pulmonary fibrosis [22]. Tran *et al*. [23] reported positive effects of adenovirus-mediated transfer of the bacterial bleomycin resistance gene in a mice model of bleomycin-induced pulmonary fibrosis. However, this approach is limited to bleomycin-induced pneumopathy, and is not useful for other types of interstitial pulmonary fibrosis. While various *in vivo *gene transfer techniques have successfully delivered genes into the lungs and treated pulmonary fibrosis [24-27], these methods are too cumbersome for standard use in fibrosis therapy. Recently, a novel variation in gene delivery was introduced wherein oligonucleotide phosphorothioates were shown to be stable in most tissues. These analogues of oligonucleotides were more resistant to nuclease than their unmodified counterparts, and were therefore expected to exhibit longer activities [28]. Without using any specific vectors, the oligonucleotides in the experiment were incorporated into alveolar cells. This approach presents a more promising method of therapy. Consequently, molecular therapy through the delivery of antisense oligonucleotides has posed a possible new treatment for pulmonary fibrosis [29].

Our study examined whether treatment with antisense oligonucleotides to HSP47 could reduce collagen deposits in a bleomycin model of pulmonary fibrosis. Our findings demonstrated that the introduction of antisense oligonucleotides against HSP47 effectively decreases HSP47 protein levels and reduces fibrotic lesions. In addition, without using any specific vectors, the oligonucleotides in the present experiment were incorporated or phagocytized into activated alveolar macrophages. Our previous study showed that *in vivo *collagen accumulation was suppressed by inhibition of its molecular chaperone in fibrotic diseases [30]. Taken together, these results suggest that antisense inhibition of the substrate-specific molecular chaperone HSP47 may provide a promising new approach to molecular therapy for pulmonary fibrosis. As a caveat, we simultaneously administered HSP47 antisense oligonucleotides and bleomycin to the rat lung in this study. We have no evidence as to the effect of delayed administration of antisense HSP47 oligonucleotides to a bleomycin affected lung, which requires further study.

## Conclusion

In this study, we demonstrate that antisense HSP47 oligonucleotides inhibited pulmonary fibrosis in bleomycin treated rat lungs. Analysis of HSP47 protein expression revealed a central role of HSP47 in the progression of bleomycin-induced pulmonary fibrosis. In fact, HSP47 protein may actively participate in the pathophysiology of pulmonary fibrosis. Consequently, HSP47 antisense oligonucleotides may be useful for treating various fibrosis.

## Abbreviations

HSP47: heat shock protein 47

CCl_4_: carbon tetrachloride

NIH: National Institute of Health

Bleo: bleomycin

AS: antisense HSP47 oligonucleotides

S: sense control oligonucleotides

H&E: hematoxylin and eosin

ECL: enhanced chemiluminescence

S.E.M.: standard error of the mean

ODN: Oligonucleotides

## Competing interests

The author(s) declare that they have no competing interests.

## Authors' contributions

SH participated in the study design, performed animal, biochemical and histological studies, and drafted the manuscript. HI planned the experimental design and performed biochemical and histological studies. SM participated in the study design and performed animal studies. TN participated in the study design, helped to draft the manuscript and coordinated the research group. All authors read and approved the final manuscript.
